# A 3-gene biomarker signature to predict response to taxane-based neoadjuvant chemotherapy in breast cancer

**DOI:** 10.1371/journal.pone.0230313

**Published:** 2020-03-20

**Authors:** Jim Kallarackal, Florian Burger, Stefano Bianco, Alessandro Romualdi, Martina Schad

**Affiliations:** OakLabs GmbH, Hennigsdorf, Germany; Biomedical Research Foundation, Academy of Athens, GREECE

## Abstract

Breast cancer is the most common cancer in women worldwide, affecting one in eight women in their lifetime. Taxane-based chemotherapy is routinely used in the treatment of breast cancer. The purpose of this study was to develop and validate a predictive biomarker to improve the benefit/risk ratio for that cytotoxic chemotherapy. We explicitly strived for a biomarker that enables secure translation into clinical practice. We used genome-wide gene expression data of the Hatzis *et al*. discovery cohort of 310 patients for biomarker development and three independent cohorts with a total of 567 breast cancer patients for validation. We were able to develop a biomarker signature that consists of just the three gene products ELF5, SCUBE2 and NFIB, measured on RNA level. Compared to Hatzis *et al*., we achieved a significant improvement in predicting responders and non-responders in the Hatzis *et al*. validation cohort with an area under the receiver operating characteristics curve of 0.73 [95% CI, 69%—77%]. Moreover, we could confirm the performance of our biomarker on two further independent validation cohorts. The overall performance on all three validation cohorts expressed as area under the receiver operating characteristics curve was 0.75 [95% CI, 70%—80%]. At the clinically relevant classifier’s operation point to optimize the exclusion of non-responders, the biomarker correctly predicts three out of four patients not responding to neoadjuvant taxane-based chemotherapy, independent of the breast cancer subtype. At the same time, the response rate in the group of predicted responders increased to 42% compared to 23% response rate in all patients of the validation cohorts.

## Background

Powerful profiling technologies and major achievements in molecular targeted therapies have triggered great expectations regarding precision medicine. However, matching patients and treatments optimally remains a pipe dream. Prerequisite for efficient precision medicine is the correct prediction of patients who will respond or not respond to a specific treatment. Current predictions mainly rely on generic biomarkers of low complexity which are used to subgroup patients of a specific indication. In breast cancer, common biomarkers are protein expression levels of estrogen receptor (ER), progesteron receptor (PR) as well as human epidermal growth factor (HER2), or mutations in the genes BRCA1 and BRCA2 [1–4]. They are associated with both prognosis and sensitivity to treatment modalities [5]. Moreover, the decision for or against chemotherapy may be guided by multigene prognostic assays such as Oncotype Dx, EndoPredict, PAM50 and BreastCancer Index, but none of them is able to guide choices of specific treatment regimes [6].

A prospective selection of patients who are most likely to respond to a given treatment is highly anticipated. Efforts are being made to develop biomarker signatures specifically for single drugs to predict pathologic complete response (pCR) or progression free survival (PFS) in contrast to residual disease (RD) after treatment. For example, Hatzis *et al*. have developed a 39-gene biomarker signature for ER-positive and a 55-gene signature for ER-negative breast cancer for taxane-antracycline-based chemotherapy in order to predict pCR [7], Horak *et al*.’s biomarker signature predicts pCR between doxorubicin-cyclophosphimide (AC)+ixabepilone vs. AC+paclitaxel [8], and Iwamoto *et al*. identified biomarker signatures that were significantly associated with pCR on F(5-fluorouracil)AC / FE(epirubicin)C treatment [9]. However, none of the proposed biomarkers converted into a companion diagnostic test for a specific chemotherapy in breast cancer.

The purpose of this study is to establish the basis for a companion diagnostic test for taxane-based chemotherapy in breast cancer patients.

Our objectives are to develop a biomarker signature of minimal size using the Hatzis *et al*. discovery cohort of 310 patients and a significant improvement of accuracy in predicting pCR in three independent validation cohorts. All objectives are factors of success in regard to translating a biomarker signature to the patients’ benefits to clinical application.

## Methods

### Patient data

We obtained four breast cancer cohorts for development and validation. In Table 1 we summarize the patients’ characteristics that were used in the present study. The discovery cohort by Hatzis *et al*. [7] of 310 breast cancer patients who received neoadjuvant taxane-antracycline chemotherapy was used for biomarker development. For validation we used in total 567 patients from three different cohorts, the validation cohort by Hatzis *et al*. [7], the Horak *et al*. cohort [10] as well as the cohort submitted by the Micro Array Quality Control consortium (MAQC) [11]. In what follows, we will refer to these as hatzis182, horak121 and maqc264, respectively. More than 95% of patients received neoadjuvant chemotherapy, the other patients received partial neoadjuvant or adjuvant chemotherapy.

**Table 1:** Breast cancer patient cohorts used for biomarker discovery and validation. Throughout this document we will use the definition of HR negative as ER negative and PR negative. While HR positive is defined as not HR negative, i.e. ER positive and PR positive, ER positive and PR negative, ER negative and PR positive.

	Discovery		Validation	
Data	Hatzis *et al*.	Hatzis *et al*.	Horak *et al*.	MAQC consortium
Name alias	-	hatzis182	horak121	maqc264
Source	E-GEOD-25055	E-GEOD-25065	E-GEOD-41998	E-GEOD-20194
Platform	HG-U133A	HG-U133A	HG-U133A_2	HG-U133A
N^(1)^	306	182	121	264
**HR status**				
positive	184	121	55	-
negative	117	60	66	-
**ER status**				
positive	172	113	45	161
negative	129	68	76	103
**PR status**				
positive	140	94	46	-
negative	160	87	75	-
**HER2 status**^(2)^				
positive	3	0	9	56
negative	288	182	112	208
**Response**				
pCR	57	42	34	55
RD	249	140	87	209
Response Rate	19%	23%	28%	20%
**Chemotherapy regime**^(3)^				
neoadjuvant	306	165	121	264
partial adjuvant	0	18	0	0
adjuvant	0	15	0	0
**Taxane**^(4)^				
Paclitaxel	287	92	121	264
Docetaxel	18	90	0	0
**Combination**				
FAC ^(5)^	227	103	0	182
AC ^(6)^	83	0	121	0
FEC ^(7)^	0	125	0	78
X ^(8)^	0	94	0	0
Trastuzumab	0	0	0	8
Other	0	0	0	5

^(1)^ Patients with reported response status were considered

^(2)^ Samples of the Hatzis *et al*. study are assumed HER2- where no explicit meta information has been found

^(3)^ Chemotherapy regime was not reported for individual patients by Hatzis *et al*.

^(4)^ Taxane was not specified for one patient in Discovery

^(5)^ Fluorouracil (F), doxorubicin (A) and cyclophosphamide (C).

^(6)^ Doxorubicin (A) and cyclophosphamide (C).

^(7)^ Fluorouracil (F), epirubicin (E) and cyclophosphamide(C).

^(8)^ Capecitabine.

### Workflow

**Fig 1:**
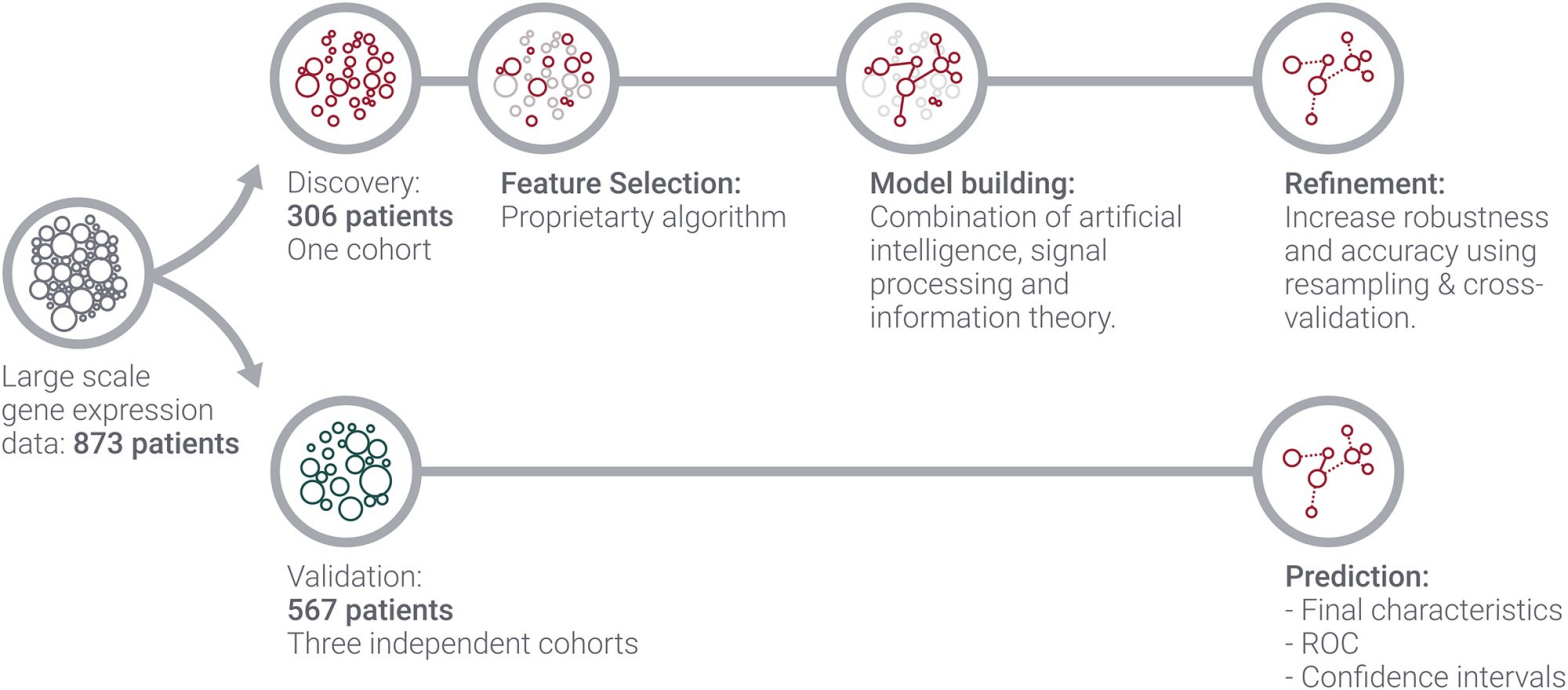
Applied biomarker workflow.

Fig 1 outlines our approach to develop and validate the predictive biomarker. Starting with genome-wide gene expression data of four independent cohorts, only one cohort was used for biomarker development. This procedure was split into three parts, feature selection, model building and refinement. Feature selection consists in reducing the data from about—in our case—20, 000 features to a small subset and was done using our proprietary algorithm. Afterwards, a model was built on the basis of the underlying genes and its parameters were tuned to maximize for best performance. In the last step, robustness and noise tolerance were determined using resampling techniques. At this stage, the classifier’s operation point was selected and fixed to be applied on the validation set.

Finally, the refined model was applied to the three independent validation cohorts with resulting receiver-operator characteristics (ROC) curves as well as performance metrics at the classifier’s operation point. We emphasize here that we emulated a prospective study as we treat each sample of the validation sets as a new instance to be classified. This requires special care when normalising microarray data to ensure that the biomarker development has no influence on the validation samples and vice versa. More details are given in section.

### Data processing and unbiased sample normalisation

Gene expression raw data were obtained from the ArrayExpress online pages (IDs: E-GEOD-25055 [12], E-GEOD-25065 [13], E-GEOD-41998 [10] and E-GEOD-20194 [11]). Both already normalised sets of data as well as raw data are provided.

The Affymetrix package (“affy”) available for the R programming language was used to normalise the raw data with the following settings: “rma” method for background correction, quantile normalisation using the “quantile” option, “pmonly” for only using the signals of the pm channel, “medianpolish” for data summary. The data set of the Hatzis *et al*. discovery cohort was normalised for biomarker development without any validation data. The parameters for the quantiles have been saved in order to treat the validation samples in the same way. For validation, each sample of the validation cohorts was quantile normalised according to the quantiles and parameters obtained from the discovery step. This approach was used to ensure that the discovery cohort is strictly separated from the validation set and that the samples of the validation sets are treated independently of each other. For the data of the Horak *et al*. cohort, the R package “inSilicoMerging” was used prior to normalising in order to adjust the different Affymetrix platform used for data acquisition. Afterwards, the Hatzis *et al*. discovery cohort and the Horak *et al*. were normalised as described above.

### Algorithms

For feature selection, we used our in-house developed proprietary algorithm on the Hatzis *et al*. discovery cohort. The algorithm attempts to identify the smallest and most robust gene set to achieve a maximum of accuracy. This is achieved by avoiding approximations wherever possible and to make use of highly parallelized computing. For classification, we have tested a variety of classification algorithms including but not limited to linear models, tree-based models (with or without boosting), different kinds of support vector machines and network-based classifiers in the discovery data set. The performance of each classification algorithm was evaluated based on the area under the curve (AUC) of the ROC curve. All models have been trained strictly on a subset of the discovery cohort of Hatzis *et al*. and their performance has been evaluated using 500 bootstrap iterations on the respective complementary set of the discovery cohort. It turned out that on the three gene set selected by our proprietary feature selection, the aforementioned classification algorithms are equally well suited to establish a reliable prediction model. For the final model, we used logistic regression. This model was fixed and used for predicting the validation data.

### ROC curve

The quantities entering the ROC computation are the probabilities to fall into either of the two classes assigned to the samples by the classification algorithm. The set of these probabilities may be considered as different prediction boundaries (classifier’s operation points) between class prediction. Each such threshold is a trade-off for the model to predict a number of true and false positives and negatives. These numbers of true and false predictions in turn convert into true positive and false positive rates that are the displayed quantities in the axes of the ROC.

We have set our classifier’s operation point to allow comparison of the performance of our biomarker with that of Hatzis *et al*. and to optimize for exclusion of non-responders. That means that the achieved sensitivity of our model matches that of Hatzis *et al*. as closely as possible. This way, the model is completely fixed and the specificity, positive predictive value (PPV) and negative predictive value (NPV) are directly comparable.

## Results

### Predictive biomarker and performance in independent validation cohorts

**Fig 2:**
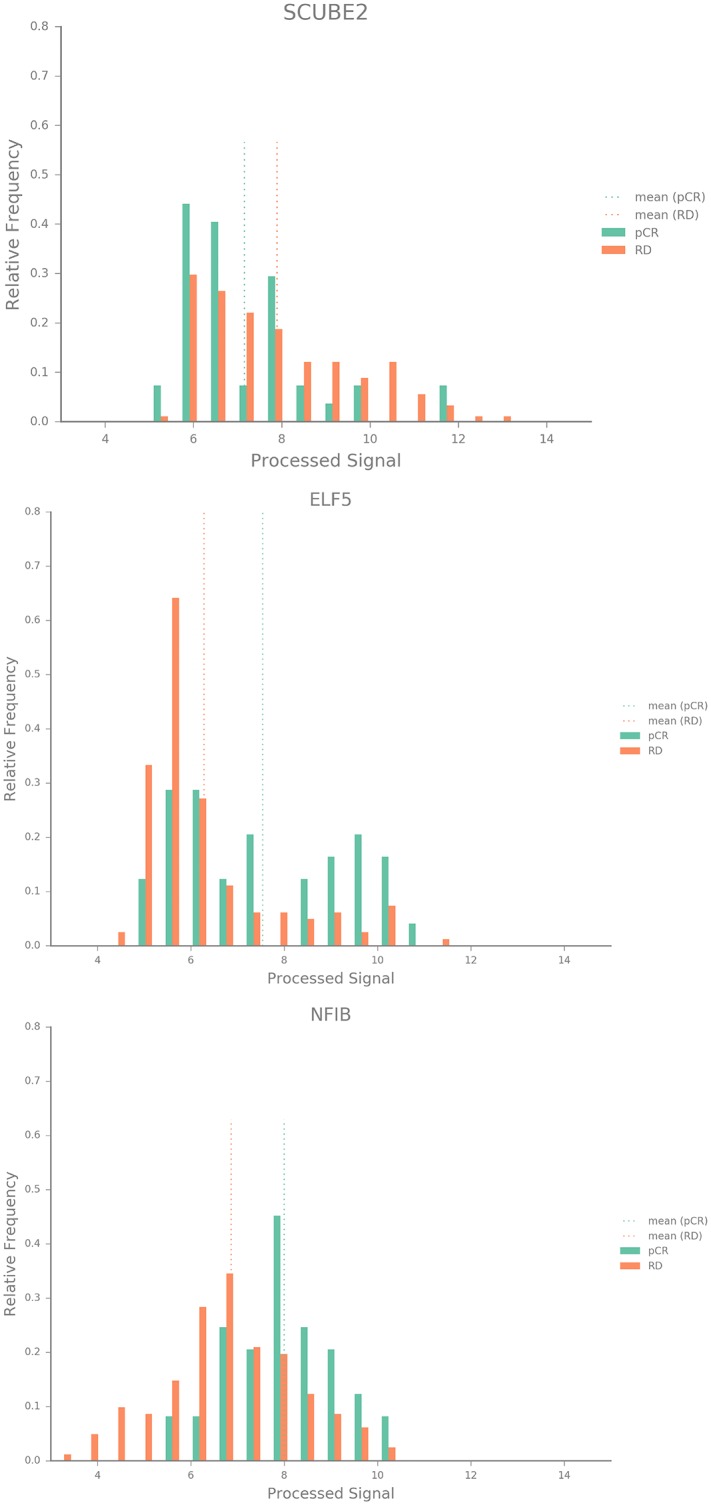
Histograms of the genes contained in the biomarker signature comparing the signal distributions within the responder class with that within the non-responder class within the validation cohort.

Using the Hatzis *et al*. discovery cohort we have developed a biomarker which consists of merely the three genes listed in Table 2. Histograms of signal distributions of the genes indicate differences in the group of responders and non-responders on gene level (Fig 2).

**Table 2:** Gene signature of our model.

Affymetrix Code	Gene Symbol
X219197_s_at	SCUBE2
X220625_s_at	ELF5
X209289_at	NFIB

**Fig 3:**
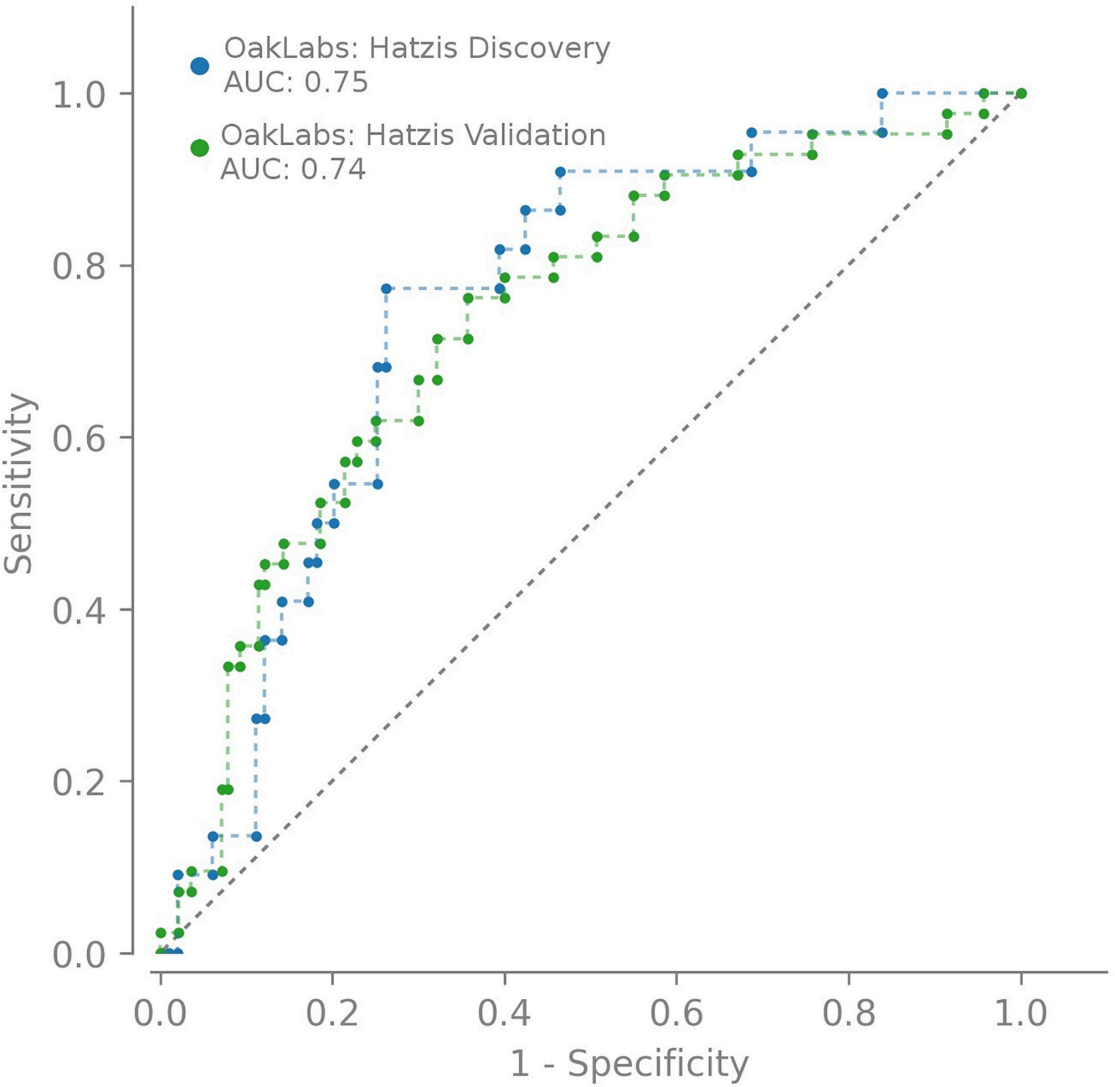
The ROC curve of our model comparing the performances on the discovery set and the Hatzis *et al*. validation set (hatzis182).

The ROC curves in Fig 3 illustrate our model’s performance on the Hatzis *et al*. discovery cohort as well as on the Hatzis *et al*. validation cohort when predicting unknown data of 182 patients. Both ROC curves are clearly compatible indicating that we have developed a valid classification model.

**Fig 4:**
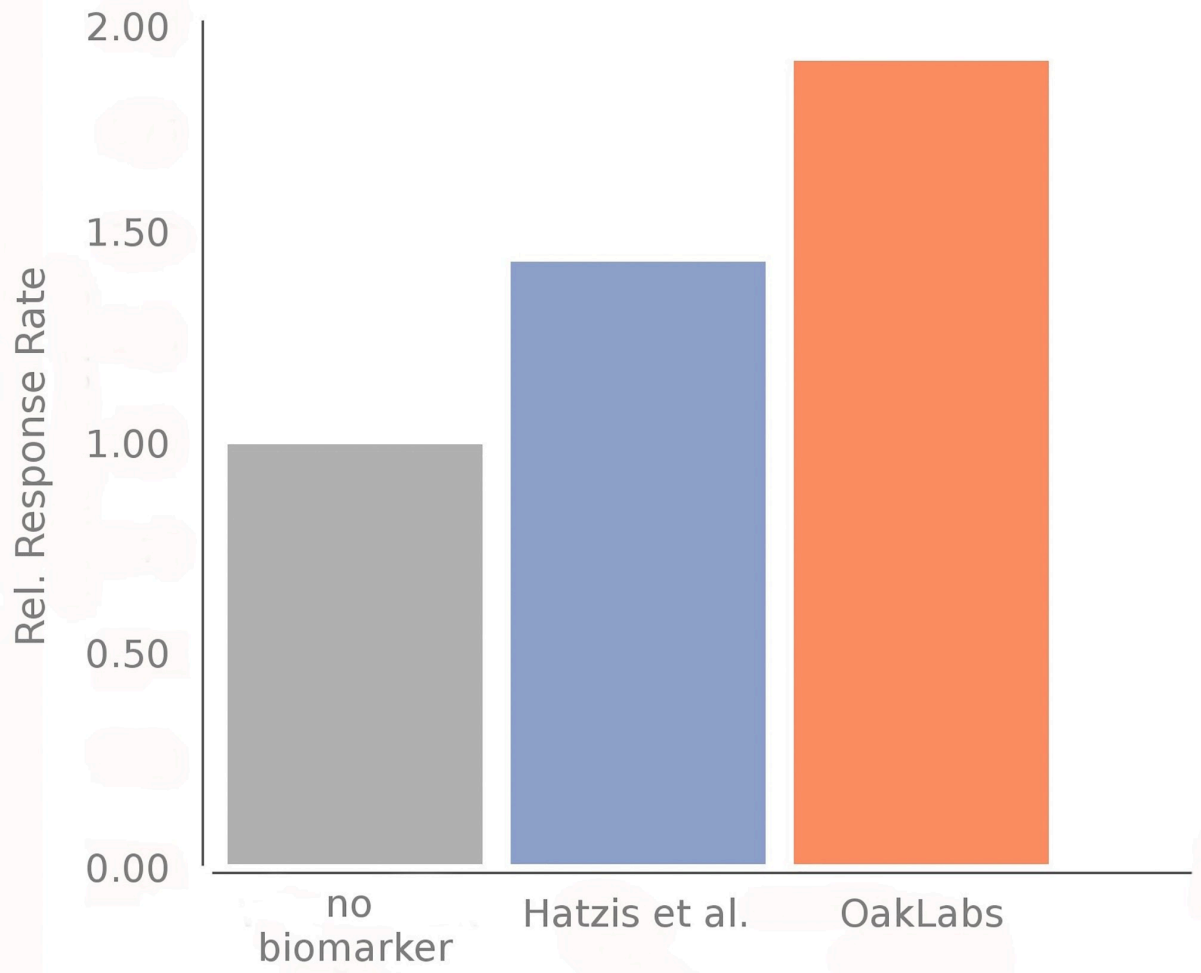
Comparison of response rate of our model to the cases without biomarker and with the model by Hatzis *et al*.

We have compared the performance of our model with that of Hatzis *et al*. [7] for predicting pCR and RD. The details on how we have deduced their performance numbers are provided in the supplementary materials. Table 3 summarizes the according model performance metrics at a classifier’s operation point which allows direct comparison of both models. That means that our achieved sensitivity of 57% matches the sensitivity of 55% by Hatzis *et al*. as closely as possible. As a PPV, which equals the response rate in the group of predicted responders, Hatzis *et al*. achieved 33%, while we obtained 44%. The improved performance is illustrated in Fig 4; response rates in the group of predicted responders are shown relative to the response rate in the whole Hatzis *et al*. cohort.

**Table 3:** Comparison of response prediction algorithm performance on the hatzis182 validation cohort (182 samples). The sensitivity of our model has been matched as closely as possible to the value of Hatzis *et al*.

	Without CDx	Hatzis *et al*.	OakLabs
Response rate	23%	33%	44%
PPV	-	33%	44%
NPV	-	83%	86%
Specificity	-	67%	79%
**Sensitivity**	-	**55%**	**57%**

We checked the performance of our biomarker on each of the three clinical sites the patients from the Hatzis *et al*. validation cohort were recruited. We observe that the prediction performance across the different sites is fully comparable which indicates the robustness of our model (S1 File).

**Fig 5:**
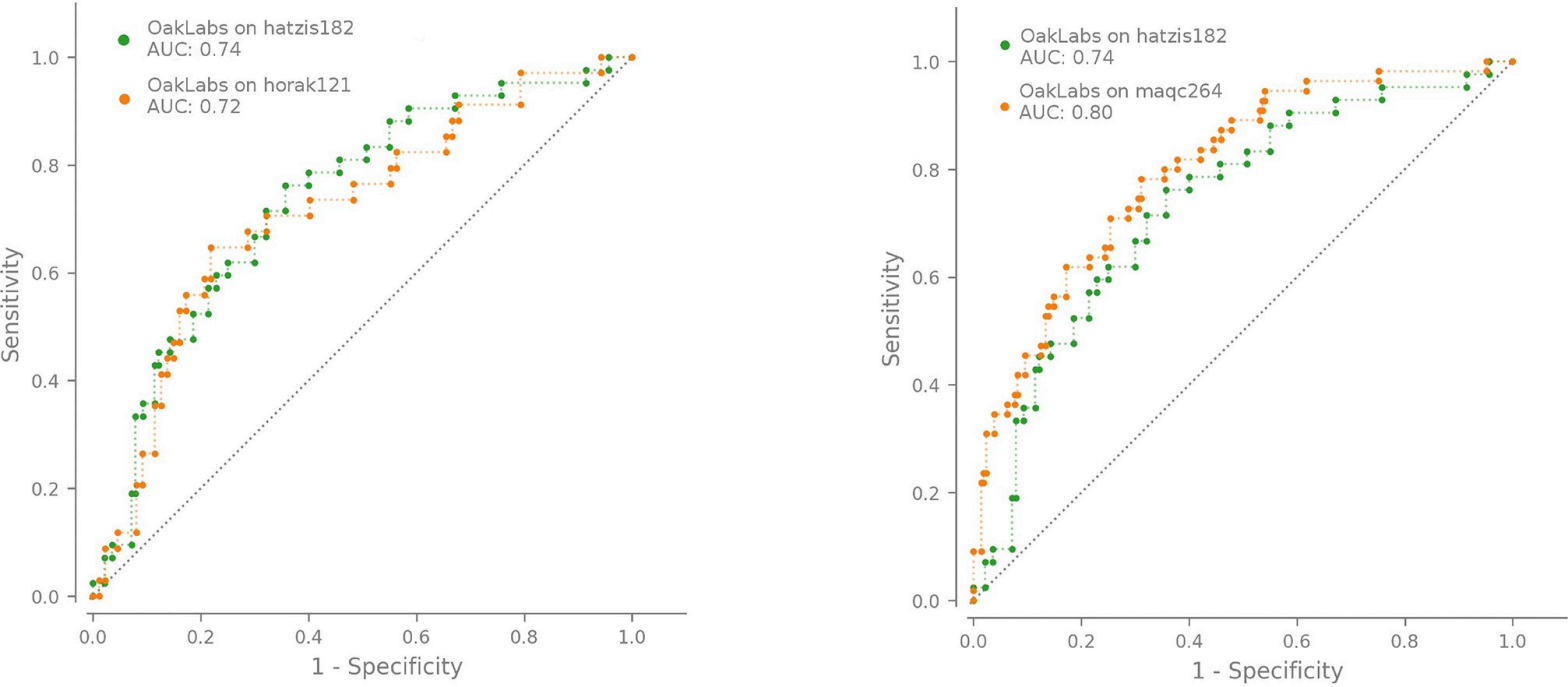
The ROC curve of our model comparing the performance of hatzis182 with horak121 (left panel) and maqc264 (right panel).

Further evidence for the reliability of our model is given by its performance in two further cohorts of in total 385 patients, the horak121 and maqc264 cohorts. The data of these two cohorts have not only been collected within different independent studies, but include also a different microarray design (horak121). We show the achieved ROC curves for the horak121 (maqc264) cohort in the left (right) panel of Fig 5, together with the ROC curve obtained with our model on the Hatzis validation set (hatzis182) for easy comparison. While the ROC curve for the horak121 cohort is comparable to the one for the hatzis182 cohort, the ROC curve for the maqc264 cohort even slightly exceeds those of the other validation sets. This is also reflected by the AUC values which are summarized together with further performance metrics in Table 4.

**Table 4:** Comparison of response prediction algorithm performance on the independent validation cohorts. Mean values together with the standard error are shown. The combined overall perfomances evaluated on all samples from the three independent validation cohorts are shown in the right-most column with the associated 95% confidence intervals.

	hatzis182	horak121	maqc264	combined
Response rate	23%	28%	20%	23%
PPV^(*)^	44(5)%	34(3)%	51(6)%	42% [37%–47%]
NPV	86(2)%	84(5)%	87(2)%	87% [84%–89%]
Specificity	78(3)%	38(5)%	87(2)%	74% [71%–78%]
Sensitivity	57(8)%	82(6)%	52(7)%	62% [53%–70%]
AUC	73(4)%	71(5)%	80(3)%	75% [70%–80%]

^(*)^ equals the response rate within the predicted responder group

The sensitivity and specificity obtained on the horak121 cohort are not in line with those of the other two cohorts. We note, however, that this behavior might be a particularity of this cohort itself which has already a higher response rate of 28% compared to 23% and 20%, respectively, in the other cohorts. For completeness, Table 4 contains as its last column the performances achieved on all 567 patients of the three independent validation data with a PPV of 42% [95% CI, 37%—47%].

Finally, we evaluated the feasibility of a single gene of our biomarker for a companion diagnostic test. ROC curves for each gene and different cohort clearly indicate that the performances of the single genes vary greatly over the cohorts. This excludes the appealing opportunity to use a single gene for a companion diagnostic test (supplementary material).

### Performance in breast cancer subtypes and different taxane drugs

The overall number of validation data from 567 patients allows to evaluate the performance of our biomarker for different subtypes of breast cancer.

**Fig 6:**
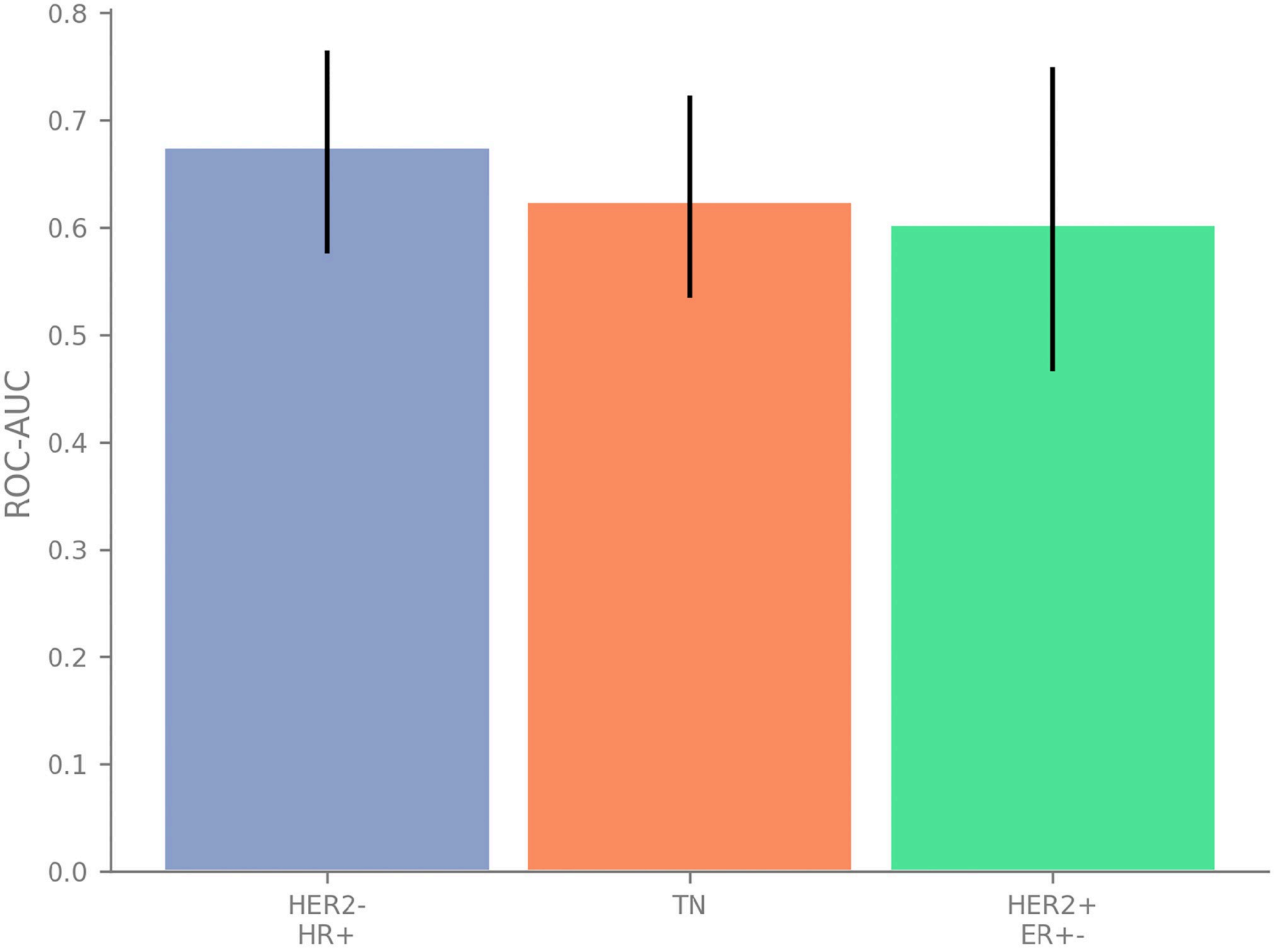
Performance summary for different breast cancer subtypes. We show the achieved ROC area under the curve mean values with the 95% confidence intervals.

To this end, we have subgrouped the validation data according to ER, PR and HER2 status and conducted a ROC AUC analysis on the stratified data. Fig 6 summarizes the AUC values with the 95% confidence intervalls obtained with our biomarker for the different suptypes.

**Fig 7:**
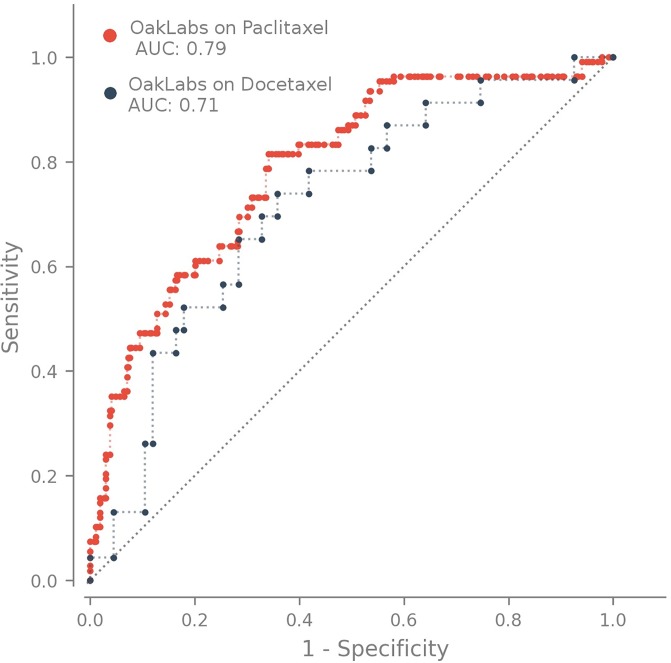
ROC curve of our model on two drugs of the taxane family, paclitaxel and docetaxel.

Finally, we have focused on the question if our biomarker works in a comparibly reliable way for different drugs of the taxane family. We tested the performance on the subgroup of patients who were treated with paclitaxel and contrasted it with the performance achieved on the docetaxel treated patient subgroup. The prediction performance distinguishing the taxane drug used in the chemotherapy is shown in Fig 7. Again we observe a similar performance in terms of the achieved ROC AUC for both taxane variants with a slightly better AUC value of 0.79 [95% CI, 0.74–0.83] for paclitaxel compared to an AUC value of 0.72 [95% CI, 0.60–0.82] for docetaxel.

## Discussion

Only a minority of breast cancer patients being treated with a cytotoxic chemotherapy actually benefits from it, while the majority is exposed to the risk of significant side effects and delay in receiving an alternative, potentially effective treatment. This does not only mean a substantial disadvantage for the affected patients’ health, but also costs the healthcare system millions of dollars each year.

In this study we strived at developing a biomarker with the capability to prevent patients not responding to a taxane-based neoadjuvant chemotherapy from receiving an ineffective treatment.

Accordingly, the classifier’s operation point of our model was chosen to optimize for exclusion of non-responders. The strict setting is supported by the availability of treatment alternatives.

The performance of our biomarker is remarkable: In a practical example it correctly predicts three out of four patients in the validation cohorts not responding to taxane-based chemotherapy. At the same time, every third patient is a predicted responder out of which 42% indeed respond to the treatment, which comprises 62% of all responders.

In a direct comparison, our biomarker outperforms that of Hatzis *et al*. by increasing the response rate to 44% in the group of predicted responders compared to the increase to 33% achieved with the Hatzis *et al*. biomarker.

Another key point is the performance of our biomarker in additional independent validation cohorts. Consistent ROC curves of all validation cohorts indicate the robustness of our biomarker. However, it has to be noted that the response rates in the group of predicted responders differ in the three cohorts, from 44% in the hatzis182 cohort, 34% in the horak121 cohort to even 51% in the maqc264 cohort. This variance might have multiple reasons. For one thing, the response rate of 28% without any response biomarker in the horak121 cohort is higher compared to that in the other cohorts. Furthermore, data of horak121 were acquired on a different technology platform.

Specifically, in regard to a CDx, we emphasize the broad applicability of our biomarker over a number of breast cancer subtypes and the major drugs of the taxane family, paclitaxel and docetaxel. Though we developed the biomarker using data from HER2- breast cancer patients only, we demonstrate significant value also for HER2+ subtypes. Furthermore, the size of the biomarker of just three genes is sufficiently small to secure translation of the biomarker into a companion diagnostics test which can easily be integrated into the routine of a clinical diagnostic laboratory.

Adding even more compelling evidence to our biomarker is that all three genes are biologically plausible. They all are described in literature in the context of cancer and breast cancer in particular. SCUBE2 (Signal peptide-complement protein C1r/C1s, Uegf, and Bmp1 [CUB]-epidermal growth factor [EGF] domain-containing protein) is a 807-amino acids protein that belongs to a small family of three members. SCUBE2 is predominantly expressed in vascular endothelial cells [14] and regulates the SHH (Sonic Hedgehog) signaling, acting upstream of ligand binding at the plasma membrane [15]. Mounting evidence suggests that SCUBE2 acts as a tumor suppressor in breast cancer [16, 17], non small cell lung cancer (NSCLC) [18], colorectal cancer [19] and gastric cancer [20]. Furthermore, SCUBE2 expression is part of the 70-gene signature (MammaPrint™) to deliver prognostic information in breast cancer patients [21].

ELF5 (E74 Like E26 transformation-specific [ETS] Transcription Factor 5) is a 265-amino acids protein and a member of the ETS family of transcription factors. ETS family proteins regulate a wide spectrum of biological processes and several ETS factors have been implicated with cancer initiation, progression and metastasis [22, 23]. For ELF5, both tumor promoting and suppressive roles have been reported in breast cancer [24]. In triple negative cancer (TNBC), high expression of ELF5 is correlated with overall survival [25].

NFIB belongs to the nuclear factor 1 (NFI) family of transcription factors which control expression of a large number of cellular genes [26, 27]. In a hetero and homodimer complex, the four members of the NFI family can activate or repress transcription depending on the context [27]. NFIB has been defined as an oncogene in several reports [28, 29]. The chromosomal region encoding NFIB is amplified in TNBC [30].

## Conclusion

Our novel biomarker robustly predicts response to taxane-based neoadjuvant chemotherapy, while outperforming the benchmark in respect to accuracy, number of features and reproducibility. The biomarker’s small size allows efficient translation to a CDx assay that is compatible with the technology in routine diagnostic laboratories.

The resulting CDx will be capable of greatly improving decision making for a specific chemotherapy and thus substantially reduce healthcare costs in breast cancer.

## Supporting information

S1 File (PDF)
