# Supplementary material for: A 3-gene biomarker signature to predict response to taxane-based neoadjuvant chemotherapy in breast cancer
Source: PLoS One. 2020 Mar 20;15(3):e0230313. doi: 10.1371/journal.pone.0230313 (PMC7083332; doi:10.1371/journal.pone.0230313)
Supplement: S1 File — (PDF) [file pone.0230313.s001.pdf]

A 3-gene biomarker signature to predict response to taxane-based chemotherapy in breast cancer.
# – supplementary material –

November 26, 2019

Jim Kallarackal, Florian Burger, Stefano Bianco,
Alessandro Romualdi, Martina Schad

1 Model Comparison Details

In order to conduct a fair model comparison between OakLabs' 3 gene signature and Hatzis *et al.*'s gene signature we have re-evaluated the available metadata of the validation cohort using the column with the prediction of "pCR" vs. "RD" of [1] (column No 20 of the sample and data relationship file). In this way we are able to directly compare two models that have been trained on the same binary categories. We considered the pCR groups as being the "positive" group and counted a sample within the pCR group that has been classified pCR as a true positive (TP) while a sample within the RD group classified as "RD" was counted as a true negative (TN). Mismatched classifications have accordingly been accounted for as false positives (FP) and false negatives (FN), respectively. Samples with missing classification in the pCR/RD column (16 in total) have been disregarded. We applied the same counting scheme for our own model, too. In this way we have computed the sensitivity (TP/(TP + FN)), specificity (TN/(TN + FP)) as well as the PPV (TP/(TP+FP)) and NPV (TN/(TN+FN)) that we report in our comparison tables in the main article.

1.1 Cross-Site Validation

In order to rule out medical site-related biases of our model we have performed a cross-site validation study. Since data taken at the MDACC site

Table 1: Cross-site study performance of our model. We show the 95% confidence intervals in parenthesis. See text for details.

Site	Sensitivity	Specificity	PPV	NPV	ROC AUC
LBJ/INEN/GEICAM	45(27)%	85(11)%	42(22)%	87(7)%	0.73
USO	64(22)%	66(16)%	51(15)%	77(11)%	0.65
MDACC	58(25)%	82(10)%	40(18)%	91(5)%	0.82
all sites	57(14)%	78(7)%	44(11)%	86(4)%	0.74

has been included both in the discovery and the validation cohort, while samples from the LBJ/INEN/GEICAM (for brevity called LBJ in what follows) and USO centers are only included in the validation cohort of the original study, the easiest way to accomplish a cross-site validation is to restrict our prediction to the latter two sites.

In this way we avoid having data originating from the same medical centers in both the learning and application stages of our model, while still being able to compare our model's performance on the validation set with values reported in literature. The site-specific performances that are achieved by our model as presented in the main text are summarized in Table 1. For reference, we have also included the performances obtained for all sites together. Additionally, we have computed the 95% confidence interval of our model performance numbers using a bootstrapping scheme over the validation cohort samples. We have found symmetrical 95% confidence intervals and report the associated errors in Table 1 in parenthesis. As can be seen, the values obtained for the USO and LBJ sites are compatible with the average obtained including all sites.

We show the achieved site-specific ROC curves in Figure 1. Comparing the AUC scores we observe that for the LBJ site (left panel) we obtain a similarly good score, while for the USO site (middle panel) we obtain a slightly worse value, which has dropped by 0.1 as compared to the discovery set. Given the typical fluctuations we observe for the AUC of about $0.04-0.06$ at the 68% confidence level (cf. the main text) the drop is insignificant. The performance on the MDACC validation set on the other hand, which we added for completeness in the right panel of the figure, is slightly better than the reference discovery value.

1.2 Single Gene Models

In Figure 2 we show the performances of models that have been trained using only a single gene of our biomarker signature. We present the ROC curves for all three validation cohorts. All single gene models show inferior

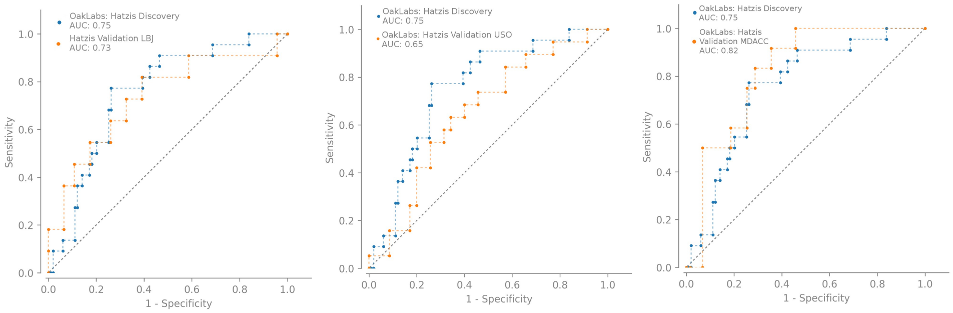

Figure 1: The ROC curves of our model comparing the site specific performances. The LBJ (left panel) and USO (middle panel) site curves demonstrate the cross site validation performance of our classification model, while the MDACC site result (right panel) is shown for completeness.

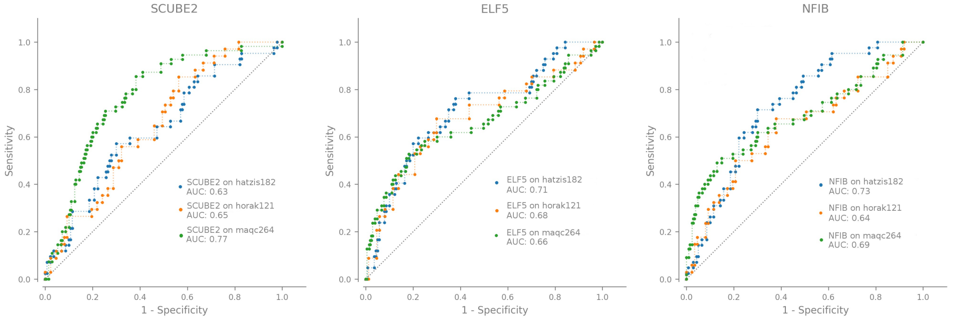

Figure 2: The ROC curves restricting the model to one single gene of the signature. In each case we show the result for each of the three validation cohorts.

prediction performances compared to our three gene model. Moreover, the variance of the performance over the individual cohorts is much larger.

References

[1] Christos Hatzis, Lajos Pusztai, Vicente Valero, Daniel J Booser, Laura Esserman, Ana Lluch, Tatiana Vidaurre, Frankie Holmes, Eduardo Souchon, Hongkun Wang, et al. A genomic predictor of response and survival following taxane-anthracycline chemotherapy for invasive breast cancer. *Jama*, 305(18):1873–1881, 2011.
